# Perforation of the bile duct caused by endoscopic papillary large balloon dilation: A case report

**DOI:** 10.1002/deo2.70015

**Published:** 2024-09-24

**Authors:** Yoichiro Sato, Naoki Okano, Kensuke Hoshi, Shuntaro Iwata, Yusuke Kimura, Susumu Iwasaki, Kensuke Takuma, Yoshinori Igarashi, Takahisa Matsuda

**Affiliations:** ^1^ Department of Gastroenterology and Hepatology Toho University Omori Medical Center Tokyo Japan

**Keywords:** bile duct perforation, bile duct stone, EPLBD

## Abstract

The patient was a woman in her 70s with multiple large biliary stones. Lithotripsy was attempted after endoscopic papillary large balloon dilatation. During balloon dilation, inflator resistance, and body movement due to patient pain were observed, and maximum pressure was required for the disappearance of the balloon waist. A bile duct perforation was observed when the balloon was deflated. Computed tomography after endoscopic retrograde cholangiopancreatography showed free air from the duodenal peritoneum to the right retroperitoneum. The patient was conservatively treated with nasobiliary drainage. Endoscopic large balloon dilatation is useful for large bile duct stones that are difficult to remove using endoscopic sphincterotomy alone or endoscopic papillary balloon dilation. Perforation is a potentially fatal adverse event; therefore, imaging of the intrapancreatic bile ducts should be performed before endoscopic retrograde cholangiopancreatography and attention should be paid to the balloon dilation method.

## INTRODUCTION

Endoscopic papillary large balloon dilatation (EPLBD) is used to dilate the biliary orifice using a large‐diameter balloon (≥ 12 mm). Erosz et al. reported the usefulness of EPLBD after endoscopic sphincterotomy (EST) for the treatment of large bile duct stones.^1^ EST plus EPLBD has been reported to be safer and more efficacious, reduces the need for mechanical lithotripters, and decreases the procedure and fluoroscopy times compared with EST alone.^2^ The early adverse events associated with EPLBD include pancreatitis, bleeding, and perforation. In a systematic review of EPLBD studies, the perforation rate was 0.6%.^3^ Most perforations improve with conservative treatment, however severe cases can be fatal. Herein, we report a case of bile duct perforation caused by EPLBD.

## CASE REPORT

**FIGURE 1:**
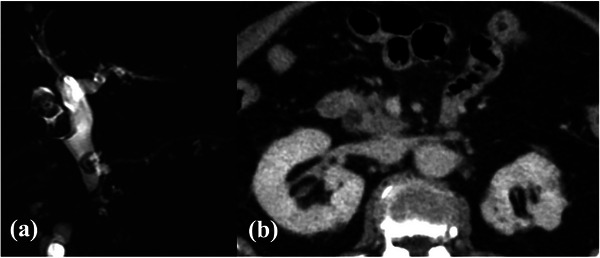
(a) Large stones at the common bile duct (13 mm) and cystic duct (16 and 18 mm). (b) The posterior wall of the intrapancreatic bile duct was only partially surrounded by the pancreatic parenchyma.

**FIGURE 2:**
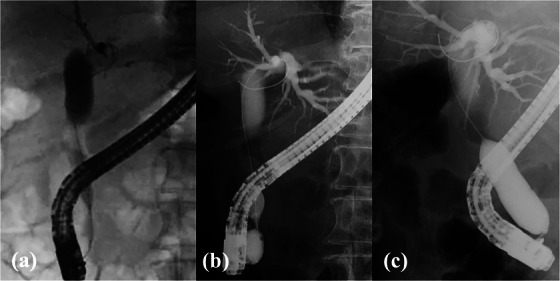
(a) Endoscopic retrograde cholangiopancreatography demonstrated a stone above a tapered distal duct. (b) Beginning of dilation with the balloon waist. (c) Full dilation was performed up to 18 mm.

**FIGURE 3:**
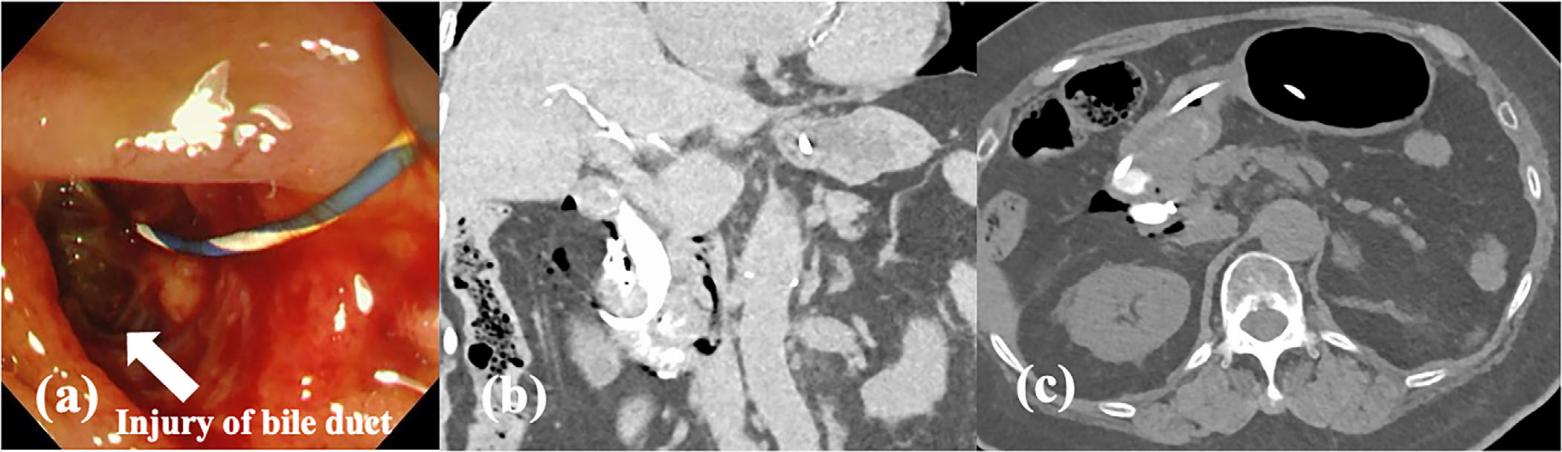
(a) Endoscopic view after endoscopic papillary large balloon dilatation showed injuries to the bile duct and hepatoduodenal mesentery from the posterior wall of the bile duct. (b, c) Computed tomography after endoscopic retrograde cholangiopancreatography showed perforation and accumulation of contrast medium at the posterior wall of the descending region of the duodenum, and free air from around the duodenum to the right retroperitoneum.

**FIGURE 4:**
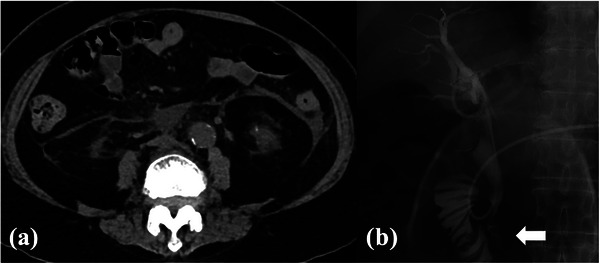
(a) Computed tomography the day after endoscopic retrograde cholangiopancreatography showed inflammatory spread to the anterior pararenal space due to pancreatitis. (b) Cholangiography from the endoscopic nasal biliary drainage tube showed a small amount of contrast medium surrounding the papilla as shown using the arrow, but no leakage into the abdominal cavity.

The patient was a woman in her 70s. Computed tomography (CT) performed at another hospital revealed a common bile duct stone (16 mm) and cystic duct stones (16 and 18 mm). The patient was referred to our hospital for further treatment. Electrohydraulic lithotripsy was planned. CT and magnetic resonance cholangiopancreatography showed a distal bile duct diameter of 16 mm, and the dorsal portion of the intrapancreatic bile duct was only partially surrounded by the pancreatic parenchyma (Figure 1). The patient had asymptomatic biliary stones, and blood tests revealed no inflammation or elevated biliary enzyme levels. ERCP was performed using an Olympus TJF‐260V video duodenoscope (Olympus) on the second day. Cholangiography revealed multiple stones and a tapered distal bile duct. The maximum bile duct diameter was 16 mm. We performed small‐EST and EPLBD using a large‐dilation balloon (REN 16–18 mm; Kaneka Medix) gradually dilated over 180 s. There was some resistance to the inflator during balloon dilation and body movements due to pain were observed when the balloon waist began to disappear. Thereafter, we continued dilating the balloon and observed the disappearance of the balloon waist at maximal pressure (8 atm, 18 mm), and the balloon deflated immediately (Figure 2). The endoscopic view of the biliary orifice after EPLBD showed bile duct injuries and hepatoduodenal mesentery from the posterior wall of the bile duct (Figure 3a). No free air was detected on fluoroscopy, and the procedure was concluded by the insertion of a nasal biliary drainage tube (endoscopic nasal biliary drainage [ENBD]). CT after ERCP revealed perforation and accumulation of contrast medium at the posterior wall of the descending region of the duodenum and free air from around the duodenum to the right retroperitoneum (Figure 3b,c). The patient complained of mild abdominal pain after the procedure. The patient was treated conservatively with fasting and fluid administration, and the abdominal pain disappeared on the third day. Peripheral blood analysis revealed a white blood cell count of 10,900 cells/µL, a C‐reactive protein concentration of 17.2 mg/dL, and pancreatic amylase activity of 137 U/L. CT revealed inflammation spread to the anterior pararenal space due to mild pancreatitis (Figure 4a). The inflammation and hyperamylasemia improved with conservative treatment. Cholangiography of the ENBD tube did not demonstrate leakage of the contrast medium into the peritoneal cavity, and the ENBD tube was removed on the eighth day (Figure 4b). The patient was started on an oral elemental diet on the ninth day, and on a diet on the 11th day. Blood test results and abdominal symptoms did not worsen after the initiation of the diet, and the patient was discharged on the 16th day. Follow‐up CT 30 days postoperatively showed fluid collection suggestive of walled‐off necrosis from the dorsal duodenum to the right anterior pararenal space. After 6 weeks of conservative antimicrobial treatment, fluid collection resolved, and the stones in the common bile duct were discharged naturally. Follow‐up CT imaging was performed for cystic duct stones (Movie).

## DISCUSSION

In cases with a dilated bile duct, EPLBD is indicated for large stones (short diameter > 12 mm) or multiple stones (> 3 stones and short diameter > 10 mm) that are difficult to remove using EST or endoscopic papillary balloon dilation alone.^4^, ^5^ EPLBD is contraindicated in patients with obvious distal bile duct strictures, a nondilated bile duct, acute pancreatitis, bleeding tendency, or those using antithrombotic medication.^4^, ^5^ Perforation is a serious adverse event that is associated with EPLBD. In an ex vivo porcine model, balloon overdilation caused bile duct perforation. However, this result is not always applicable to humans, as the bile duct wall of pigs is thin, and the pancreatic parenchyma does not surround the lower bile duct.^6^ Results from a multicenter study showed that distal bile duct stricture was a risk factor for perforation, independent of the presence of EST or balloon diameter.^7^ EPLBD is not recommended for nondilated or tapered distal bile ducts. However, there are no definite criteria for measuring the diameter of the bile duct or a consensus on a suitable diameter for EPLBD. Selection of a balloon size that does not exceed the diameter of the stone or the distal bile duct diameter is advocated. There is no clear evidence of an association between the balloon diameter and the frequency of procedural accidents. Dilation that does not exceed the distal bile duct diameter is recommended. Therefore, an appropriate balloon size should be chosen when attempting EPLBD. Regarding the speed of dilation, the results of animal studies advocate gradual dilation to prevent perforations associated with overdilation. There is no obvious evidence of the effect of dilation duration which varies from 30 s to 6 min, depending on the report. There are reports on the usefulness of prolonged dilation, but there seems to be no difference in the success rate of stone removal or the incidence of adverse events based on the duration of dilation. Safe methods of balloon dilation to prevent perforation include dilating the balloon after pushing the lower bile duct stone upstream, not applying additional pressure if strong resistance of the inflator is encountered during balloon dilation, and immediately deflating the balloon when the patient feels pain.^7^ Some reports have suggested that, if the balloon waist does not disappear after dilation to 75% of the manufacturer's recommended maximum inflation pressure, further dilatation is not recommended because of the risk of perforation.^8^ The anatomy of the intrapancreatic bile duct should be evaluated using preprocedural imaging because the distal bile duct may run outside the dorsal pancreatic parenchyma and may be partially surrounded by the parenchyma due to pancreatic atrophy or a duodenal diverticulum. Recently, there have been reports on the safety of EPLBD for distal bile ducts without bile duct dilatation. Studies have shown that overdilation is not a risk factor for perforation if the pancreatic parenchyma surrounds the bile duct.^9^, ^10^ However, some reports have suggested that the use of balloons ≥ 16 mm may cause a high rate of adverse events. Several studies have reported on the usefulness of peroral cholangioscopy (POCS)‐guided lithotripsy. Considering the adverse events, we believe that crushing stones with POCS‐guided lithotripsy and removing them with minimal balloon dilation is safer than overdilating or attempting to remove stones without crushing them after EPLBD. This report describes a case of a perforation caused by EPLBD. We believe that the anatomical characteristics of the intrapancreatic bile duct, pain and resistance of the inflator during balloon dilation, and 18 mm balloon overdilation for a bile duct diameter of 16 mm contributed to the perforation. When attempting EPLBD, an appropriate balloon dilation that does not exceed the diameter of the bile duct is important. When a perforation occurs, it is important to prevent bile and pancreatic juice leakage after the procedure. The effectiveness of a fully covered metallic stent placement for bile duct perforation has been previously reported. As no free air was observed on fluoroscopy, we considered a small perforation and placed an ENBD tube instead of a fully covered metallic stent. Pancreatic duct drainage should have been considered in the context of pancreatitis and fluid retention caused by the leakage of pancreatic juice. This patient improved with conservative treatment, but in cases of poorly controlled biliary fistula, pancreatic fistula, retroperitonitis, or pancreatitis due to persistent leakage of bile and pancreatic juice from the perforation site, the placement of a fully covered metallic stent or surgical treatment should be considered. In the future, definite criteria for the indication of bile duct for EPLBD and dilation methods should be established.

## CONFLICT OF INTEREST STATEMENT

None.

## Supporting information

Endoscopic view after endoscopic papillary large balloon dilation (EPLBD) showing bile duct perforation.
